# Portable-size artificial uterine system for viviparous shark embryos

**DOI:** 10.1016/j.mex.2024.103063

**Published:** 2024-11-17

**Authors:** Taketeru Tomita, Atsushi Kaneko, Minoru Toda, Hiromi Morota, Kiyomi Murakumo, Keiichi Sato

**Affiliations:** a Okinawa Churashima Research Institute, Okinawa Churashima Foundation, Motobu, Japan; b Okinawa Churaumi Aquarium, Okinawa Churashima Foundation, Motobu, Japan; c Okinawa Churashima Foundation Veterinary Hospital, Okinawa Churashima Foundation, Motobu, Japan

**Keywords:** Portable-size artificial uterus for viviparous shark embryo, Artificial womb, Artificial uterine fluid, Conservation breeding, Elasmobranchs

## Abstract

In this study, we developed a portable artificial uterus specifically designed for viviparous elasmobranchs (sharks and batoids). This new method is different from the previous method owing to the absence of fluid-cleaning filters, a smaller amount of incubation fluid, and the use of a mini-sized refrigerator for temperature control. Due to these modifications, the total weight decreased to approximately 40 kg, which is less than one-twentieth that of the previous system. We confirmed that embryonic lanternsharks (*Etmopterus molleri*) survived in this system for over two months, demonstrating its efficacy. In addition to its primary use as an extra-uterine life support system for viviparous elasmobranch embryos in laboratory conditions, the new system can be used for the long-term transport of premature embryos, which was not feasible with previous systems.

• A novel artificial uterine system for viviparous elasmobranchs was developed.
• The new system is reduced in size to a portable level by simplifying the previous system.
• This system can be used for long-term transport of prematurely born embryos.

## Graphical abstract

**Figure:**
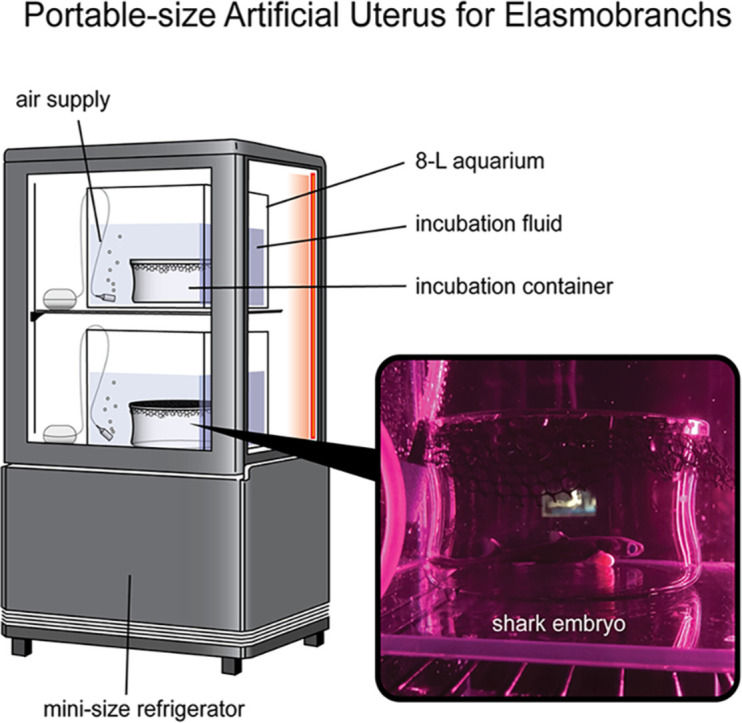

Specifications table

**Table:** 

Subject area:	Veterinary Science and Veterinary Medicine
More specific subject area:	Artificial uterine science
Name of your method:	Portable-size artificial uterus for viviparous shark embryo
Name and reference of original method:	Not applicable
Resource availability:	Not applicable

## Background

Artificial uterus is a medical technology that supports the life of immature embryos/fetuses of viviparous animals outside the maternal body. Such technology has been developed mainly for mammals, aiming for future applications in humans, but few attempts have been made for non-mammalian vertebrates, including elasmobranchs (sharks and batoids) [1]. We recently developed a novel artificial uterine system for elasmobranchs, characterized by using a urea-containing solution with osmotic pressure and salinity nearly equivalent to shark blood plasma for embryonic incubation (“artificial uterine fluid”). Using this fluid, we successfully maintained embryonic lanternsharks for maximally one year until birth size was reached at the Okinawa Churaumi Aquarium (Okinawa, Japan) [2,3,4]. Such a study is helpful in the medical treatment of prematurely born embryos for captive individuals in public aquaria and in conservation breeding for wild populations of threatened species.

Despite these technological advances, the current system is limited by its low portability. In addition to the incubation chamber for embryos, the current system has a large life-support system, including fluid cleaning filters, a fluid circulation system, a respiratory air supply, and temperature control, resulting in a total weight of >1000 kg [2]. Such a large size makes transport practically impossible; thus, the application of this system has been limited to populations of elasmobranchs that are distributed relatively close to the laboratory where the artificial uterine system is installed (e.g., where the transport time of the embryos is only a few hours). To solve this problem, we developed a new system that greatly simplifies the previous system and thus reduces its size to a portable level.

## Method details

**Fig. 1:**
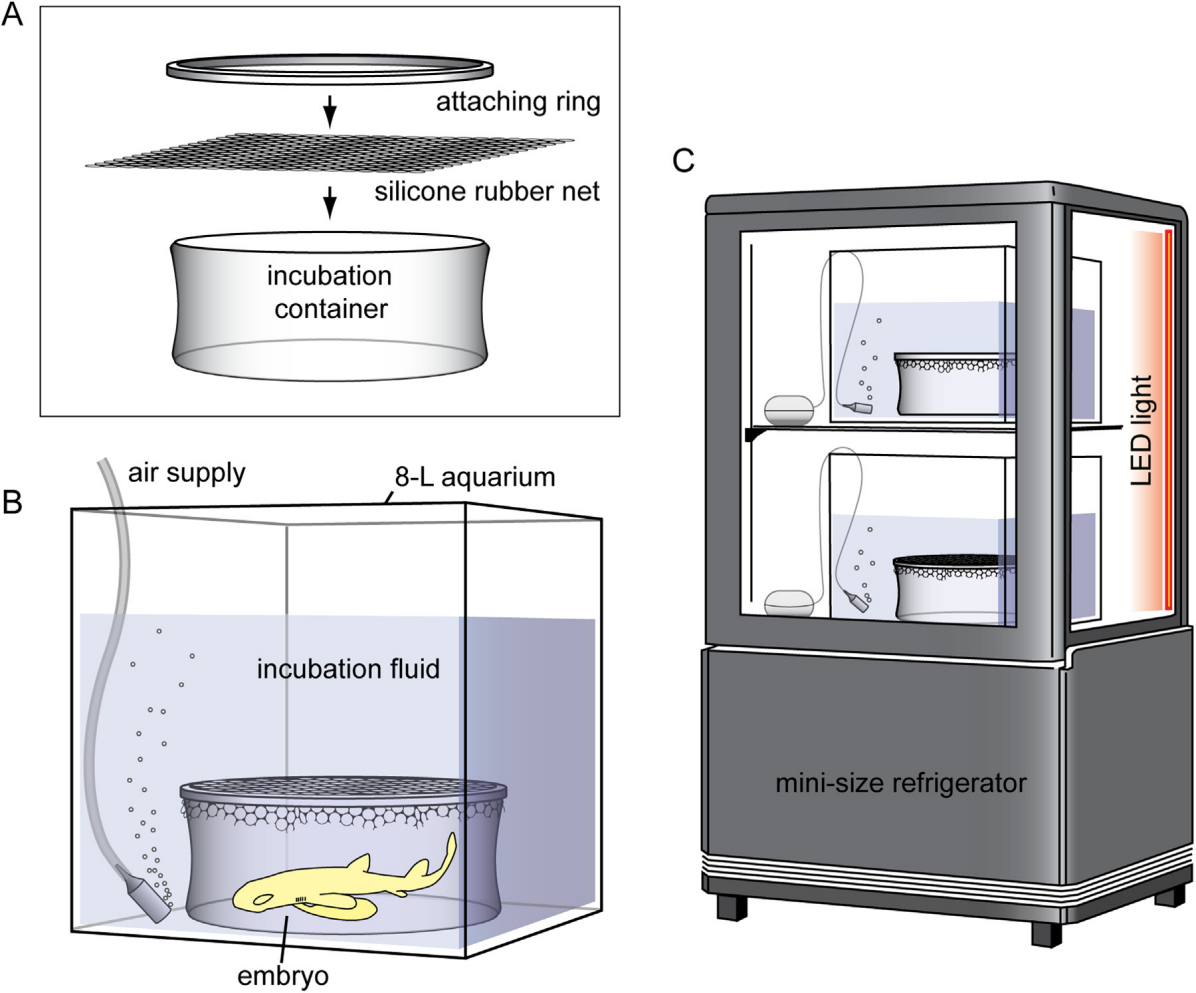
Artificial uterine system developed in this study. **A**. The incubation container is covered with a silicone rubber net. **B**. 8-L aquarium contains 5-L incubation fluid, and the incubation container is placed in the aquarium. **C**. Two 8-L aquariums, including embryos, are placed in the mini-size refrigerator for temperature control.

The entire system is illustrated in Fig. 1. Two 8-L cubic glass aquariums were placed in a mini-size refrigerator, R4G-63SLB (Remacom Co., Ltd., Shizuoka, Japan; internal volume = 63 L), for temperature control. Each aquarium includes 5-L incubation fluid (see construction details in [2] and [3]). A small cylindrical glass container with a diameter of 13 cm and a height of 6.5 cm was placed on the floor of each aquarium and used for embryonic incubation. The top of the container was covered with a silicone rubber net with a mesh size of 5 mm, allowing for the free exchange of fluid between the inside and outside of the container. Each container contained a single embryo. Air was supplied near the bottom of each aquarium and outside the container. The entire system was maintained in the dark. For embryonic observation, LED lights were placed in a refrigerator and turned on as needed. The LED lights were covered with a red plastic film to minimize light stress on the embryos.

## Method validation

### Materials and methods

A single pregnant slender lanternshark (*Etmopterus molleri*) was obtained from local fishermen at a depth of approximately 500 m, off the main island of Okinawa on December 8th, 2023. Fish were captured using hook-and-line fishing and maintained in a cooler box filled with seawater in a fishing boat. This specimen died during the 6-h return trip and was donated to the Okinawa Churaumi Aquarium (Okinawa, Japan) on the same day of capture. In the aquarium, we dissected the specimen and recovered six embryos from the uterus. Immediately after dissection, the embryos were introduced into an artificial uterus. The condition of the embryo in the artificial uterus was visually assessed twice a day (9:00 and 17:00) by observing respiratory behavior (e.g., movement of gill flaps and spiracular valve) and response to light stimuli (e.g., body undulation and changing body posture). The fluid temperature was maintained at 12 °C throughout the incubation period, which was close to that of the natural habitat of the species.

The embryos were maintained in the artificial uterine fluid, which was developed as described in a previous study [2]. This fluid was prepared by dissolving 3.5 kg of urea in 46 L of chlorine-free tap water, which was made up to a final volume of 100 L using filtered seawater. Through incubation, the chemical composition of the fluid gradually shifted away from that of the artificial uterine fluid and into full-strength seawater in a stepwise manner. This allowed the embryo to acclimate to the seawater environment. During this acclimation process, the incubation fluid was prepared by mixing artificial uterine fluid and seawater while changing the mixing ratio following the protocol developed in a previous study [3]: 100/0, 75/25, 50/50, 25/75, 12.5/87.5, and 6.3/93.7. The interval between each step of chemical composition shift was approximately one week.

**Fig. 2:**
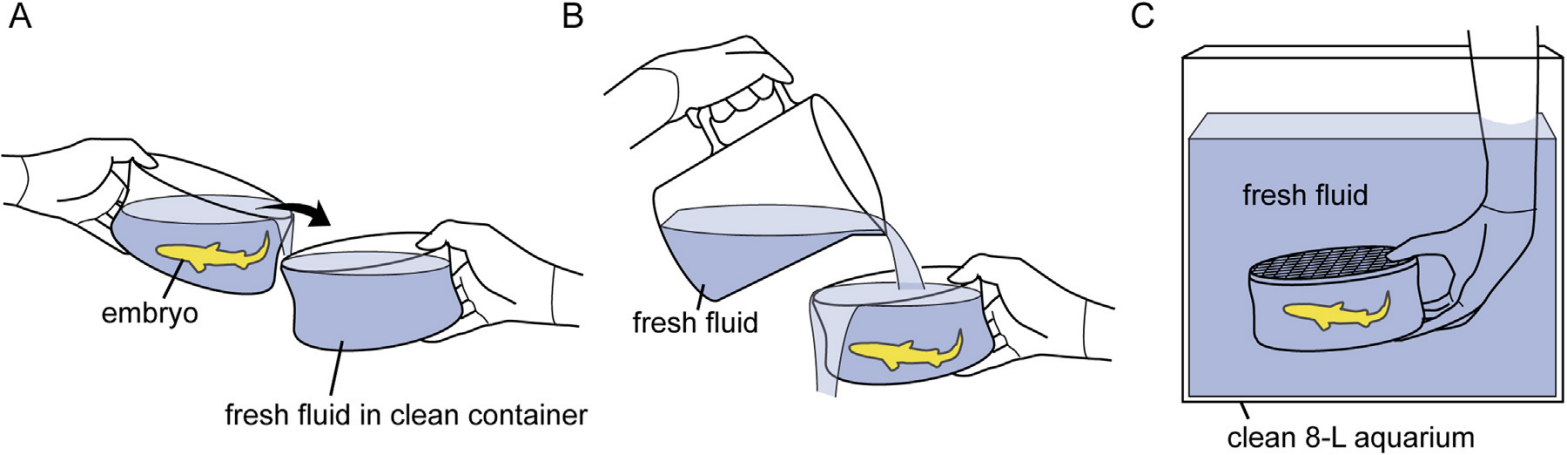
Protocol for fluid replacement. **A**. The embryo was moved to a clean container filled with fresh incubation fluid. **B**. Briefly, 2 L of fresh incubation fluid was poured into the container to wash the body surface of the embryo. **C**. The container, including the embryo, was placed in a clean 8-L aquarium filled with fresh incubation fluid. The aquarium was then returned to the refrigerator.

The chemical composition of the incubation fluid was monitored every two–four days for sodium and urea and daily for ammonia using a DRI-CHEM NX600 automated clinical chemistry analyzer (Fujifilm Co., Tokyo, Japan). Because the ammonia concentration increased through time, the incubation fluid was replaced with new fluid trying to keep the ammonia concentration under 200 µg/dL. Fluid replacement for each embryo was conducted as follows: first, the embryo in the incubation container was moved to a clean container filled with fresh incubation fluid (Fig. 2A). Second, an additional 2 L of fresh incubation fluid was poured into the container to wash the body surface of the embryo (Fig. 2B). Third, the container with the embryo was placed in a clean 8-L aquarium filled with fresh incubation fluid and returned to the refrigerator (Fig. 2C). The used aquarium and container were washed with 0.1 % sodium hypochlorite solution, rinsed with tap water, dried, and used in the next fluid replacement.

## Results

**Fig. 3:**
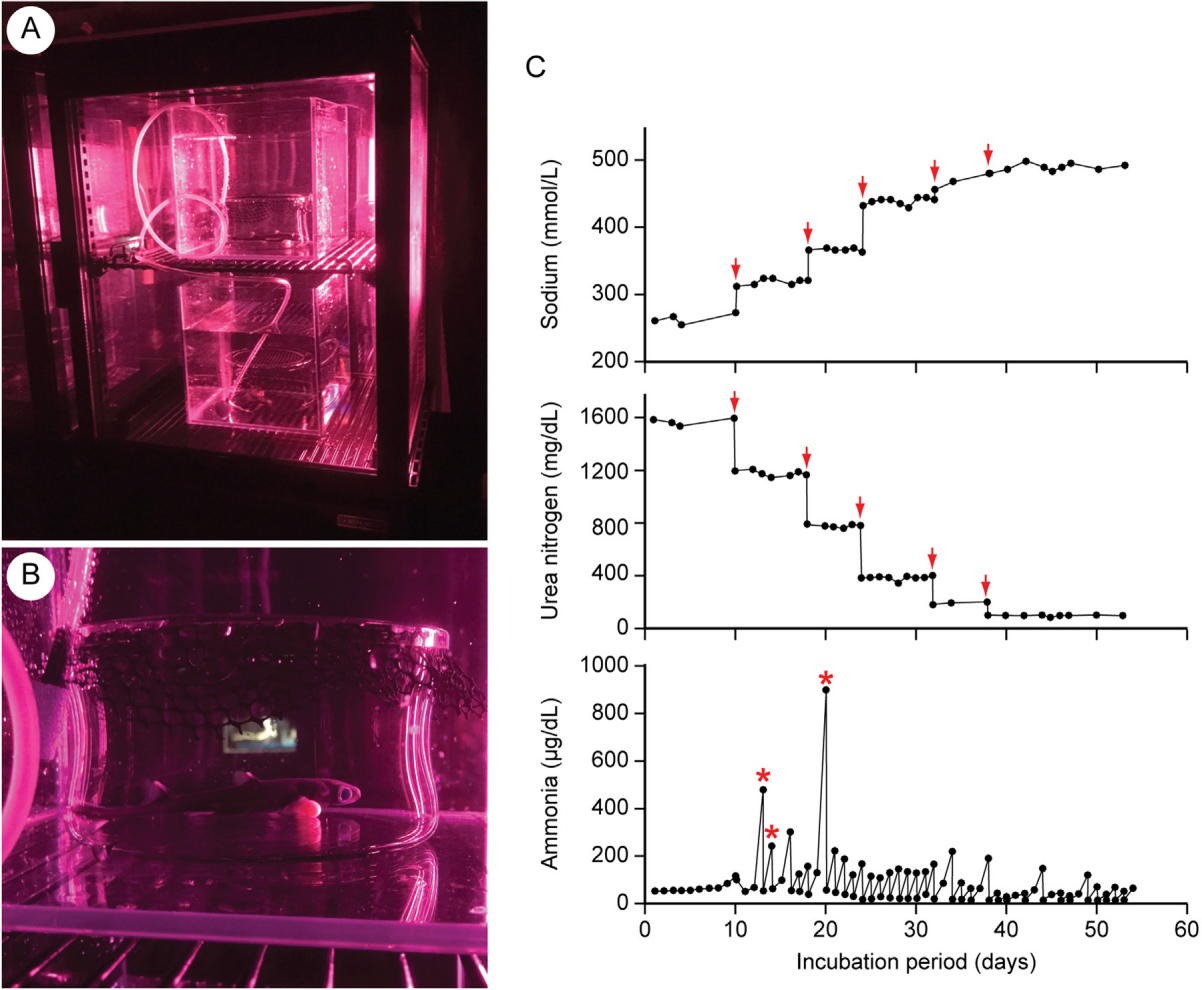
Application of the new artificial uterine system to the embryos of the slendertail lanternshark (*Etmopterus molleri*). **A**. Photograph showing the embryonic incubation of the slendertail lanternshark. **B**. Closeup view of the incubated embryo shown in A. Yellow external yolk-sac is attached to the body of the embryo. **C**. Change in the concentrations of sodium (top), urea (middle), and ammonia (bottom) of the incubation fluid. Red arrows shown in the top and middle graphs show the timing of the changing of the fluid chemistry. Red asterisks shown in the bottom graph show the rapid increase of ammonia after embryonic defecations.

Five of the six embryos successfully grew in the artificial uterus for a maximum of 77 d until they reached birth size (approximately 15 cm in total length) (Fig. 3A, B). The remaining one embryo died on the 31st day of incubation, probably due to the bacterial infection caused by the skin abrasion of the snout. When the external yolk sac was completely absorbed, five live embryos were sequentially moved from the artificial uterus to a 500-L seawater tank located outside of the artificial uterus (= “delivery”). They were fed a meal comprising a mixture of minced chub mackerel, *Scomber japonicus*, and minced sakura shrimp, *Lucensosergia lucens*, on the eighth day after “delivery.” The feeding protocol was based on [3].

The change in the chemical composition of the incubation fluid over time is shown in Fig. 3C. During the incubation period, ammonia concentrations were kept mostly below 200 µg/dL with only manual fluid replacements (Fig. 3C). The daily increase in ammonia concentration remained at a very low level of less than 10 µg/dL for the first 8 days, but then increased to about 100 µg/dL. Because of this change, the incubation fluid was manually changed almost daily after 8 days. In addition to these “regular” ammonia increases, we detected some unexpected increases after embryo defecation (red asterisks in the lower graph of Fig. 3C). This type of ammonia increase was so rapid that the daily increase reached 776 µg/dL on day 19 of incubation.

## Comparison to the previous method and future applications

The artificial uterine system presented in this study is clearly distinguished from previous systems by the absence of fluid-cleaning filters, a smaller amount of incubation fluid, and the use of a mini-sized refrigerator for temperature control. Owing to these modifications, the total weight of the system is approximately 40 kg, which is less than one-twentieth that of the previous system described in [2]. Such a size reduction allowed the artificial uterus to be used for portable purposes, such as carrying on boats and land vehicles with a 100 V electric supply from portable generators or batteries.

The small amount of fluid in the new system also requires less effort for fluid replacement. The previous system required 6 h and at least three persons for each fluid replacement, whereas the new system, only required 2 h and one person. However, it should be noted that the new system requires fluid replacement more frequently (approximately two times more often) than the previous method. Generally, relatively minor environmental disturbances easily affect filterless and small-sized systems, making the internal environment less stable. The ammonia concentration of the incubation fluid was greatly increased by embryonic defecation in the new system, although this phenomenon was not observed in the previous systems [[2], [3], [4]].

Considering these advantages and disadvantages, the new system is well suited for the temporary maintenance of embryos, especially during embryo transport from the wild into a laboratory environment, where more reliable stationary artificial uterine systems are installed. Establishing such an embryo transport method would significantly expand the spatial limits to which artificial uterine technology can be applied.

## Limitations

This method can be used for elasmobranchs with yolk sac viviparity, characterized by embryonic development depending solely on the yolk. In contrast, this method cannot be applied to other viviparous modes (e.g., lipid histotrophy) in which the mother supplies additional nutrition.

## Ethics statements

The handing of the animal was done in strict accordance with the guidelines for animal experiments of the Okinawa Churashima Foundation, with the same consideration for animal care and welfare as that for “higher” vertebrates (reptiles, birds, and mammals). As the guidelines stipulate, the approval from the Institutional Animal Care and Use Committee of Okinawa Churashima Foundation, required for higher vertebrates, is waived for “lower” vertebrates, including fishes.

## Supplementary material *and/or* additional information [OPTIONAL]

Not applicable.

## CRediT authorship contribution statement

**Taketeru Tomita:** Conceptualization, Formal analysis, Investigation, Data curation, Writing – original draft. **Atsushi Kaneko:** Conceptualization, Methodology, Investigation. **Minoru Toda:** Conceptualization, Methodology, Investigation. **Hiromi Morota:** Investigation. **Kiyomi Murakumo:** Methodology. **Keiichi Sato:** Supervision, Writing – review & editing.

## Declaration of competing interest

The authors declare that they have no known competing financial interests or personal relationships that could have appeared to influence the work reported in this paper.

## Data Availability

Data will be made available on request.

## References

[1] Otway N.M., Ellis M.T. (2012). Construction and test of an artificial uterus for *ex situ* development of shark embryos. Zoo Biol..

[2] Tomita T., Toda M., Murakumo K., Kaneko A., Yano N., Nakamura M., Sato K. (2022). Five-month incubation of viviparous deep-water shark embryos in artificial uterine fluid. *Front. Mar. Sci.*.

[3] Tomita T., Toda M., Kaneko A., Murakumo K., Miyamoto K., Sato K. (2023). Successful delivery of viviparous lantern shark from an artificial uterus and the self-production of lantern shark luciferin. PLoS One.

[4] Tomita T., Toda M., Kaneko A., Murakumo K., Miyamoto K., Sato K. (2024). One-year extra-uterine life support for viviparous shark embryos: first technological application to mid-term embryos. *Front. Fish Sci.*.

